# Clinical Evidence of Acetyl-L-Carnitine Efficacy in the Treatment of Acute Ischemic Stroke: A Pilot Clinical Trial

**DOI:** 10.1155/2022/2493053

**Published:** 2022-07-29

**Authors:** Mehrdokht Mazdeh, Parnaz Abolfathi, Maryam Sabetghadam, Younes Mohammadi, Maryam Mehrpooya

**Affiliations:** ^1^Department of Neurology, School of Medicine, Hamadan University of Medical Sciences, Hamadan, Iran; ^2^Department of Clinical Pharmacy, School of Pharmacy, Medicinal Plants and Natural Products Research Center, Hamadan University of Medical Sciences, Hamadan, Iran; ^3^Modeling of Noncommunicable Diseases Research Center, School of Public Health, Hamadan University of Medical Sciences, Hamadan, Iran

## Abstract

**Background:**

This study was undertaken to evaluate the influence of oral Acetyl-L-carnitine (ALC) in patients with acute ischemic stroke.

**Methods:**

Sixty-nine cases with acute ischemic stroke with the onset of symptoms less than 24 hours not candidates for reperfusion therapy were randomly assigned to either the ALC group (1000 mg three times per day for three consecutive days) or the matching placebo group. The study outcomes based on intention-to-treat criteria included the change in the modified Rankin Scale (mRS) and National Institutes of Health Stroke Scale (NIHSS) score from baseline to day 90, as well as the change in serum levels of the inflammatory and oxidative stress biomarkers over the 3-day treatment protocol.

**Results:**

The NIHSS score and mRS score on day 90 were improved by 5.82 and 0.94 scores, respectively, in the ALC-treated group compared to 2.83 and 0.11 scores, respectively, in the placebo-treated group, which demonstrated the superiority of ALC relative to placebo. By using the multivariable analysis after adjusting for other variables in the model, compared to the group treated with placebo, patients in the ALC group had lower NIHSS score (*β*: -2.40, 95% CI: -0.69, -4.10 (*p* = 0.007)) and mRS score (*β*: -1.18, 95% CI: -0.52, -1.84 (*p* = 0.001)) 90 days after the intervention. The percentage of patients with a favourable functional outcome at day 90, defined as mRS scores of 0 or 1, was significantly higher in the ALC group in comparison to the placebo group (52.9% versus 28.6%). Further, over the 3-day treatment protocol, in the patients receiving ALC, the serum levels of proinflammatory biomarkers, including soluble intercellular adhesion molecule-1 (sICAM-1), interleukin 6 (IL-6), tumor necrosis factor-alpha (TNF-*α*), and neuron-specific enolase (NSE), showed a significant decrease, while the serum levels of antioxidant biomarkers, including glutathione peroxidase (GPx), superoxide dismutase (SOD), and total antioxidant capacity (TAC), as well as the total L-carnitine's level showed a significant increase compared to those in patients receiving placebo indicating significant alteration.

**Conclusions:**

Although preliminary, these results suggested that ALC administration during the acute phase of ischemic stroke might be helpful in improving functional and neurological outcomes that are probably linked to its anti-inflammatory and antioxidant properties. *Trial Registration*. This trial is registered with IRCT20150629022965N17 at Iranian Registry of Clinical Trials (registration date: 25/07/2018).

## 1. Introduction

Despite advances in identifying and reducing the risk, stroke remains among the leading causes of death and adult-acquired disability throughout the world [1], as up to 45% of patients with stroke do not expect to recover independence [2]. Ischemic stroke, which constitutes about 80 to 85% of all strokes, stems from an embolic or atherothrombotic obstruction to a cerebral artery [3]. Although thrombolysis with alteplase is the only available pharmacological agent for the treatment of acute ischemic stroke, this treatment is applicable only for a limited number of patients with ischemic stroke due to its very short duration (4.5 hours) and concerns about the increased risk of intracerebral hemorrhage [4]. Thus, it is imperative to develop novel therapeutic strategies for stroke, particularly for those cases lacking eligibility for thrombectomy or thrombolysis.

Strong evidence indicates that oxidative stress and inflammation are two of the fundamental pathophysiological mechanisms of cell damage following cerebral ischemia [5]. Because of the limited antioxidant defense of the brain, it is highly vulnerable to radical-mediated attack. Therefore, increasing cellular generation of free radicals, particularly reactive oxygen species (ROS), early after ischemia by injury to cellular compounds, including protein, nucleic acids, and lipids, can ultimately lead to cell injury [6]. In addition to its direct cellular injury, oxidative stress indirectly via stimulating the generation of proinflammatory cytokines, like tumor necrosis factor-*α* (TNF-*α*), interferon-*γ*, interleukin-1 (IL-1), and IL-6 by leukocytes, microglia, and resident cells of the neurovascular unit, results in worsening of cerebral ischemic damage [7].

Neuroinflammation is another important pathological pathway involved in the pathogenesis of ischemic brain damage after a cerebrovascular attack. Although inflammatory responses in the later phases of the stroke injury process may correspond to brain repair and recovery after stroke [8], robust inflammatory responses a few hours after the onset of stroke contribute to ischemic brain injury [9]. Releasing various proinflammatory mediators, like chemokines, cytokines, matrix metalloproteinases (MMPs), and cell adhesion molecules (CAMs) immediately after the onset of ischemia through direct and indirect mechanisms, can aggravate neuronal damage and death [5]. It is noteworthy that the magnitude of plasma levels of these proinflammatory mediators has shown a correlation with early neurological deterioration and poor functional outcomes after stroke [10]. On the flip side, a wealth of literature from experimental studies demonstrates that suppressing the actions of these proinflammatory mediators can decrease the infarct size and has a neuroprotective effect against ischemic brain injury [11–13].

Supporting this evidence, large numbers of experimental and human-based studies have shown the beneficial effects of early treatment with anti-inflammatory and antioxidant compounds in the improvement of the clinical outcomes of patients with stroke [14, 15]. L-Carnitine and its derivatives also can be candidate molecules for targeting these pathological pathways. L-Carnitine is a quaternary amine produced in the liver, kidneys, and brain tissue and is available in tissues of most mammalians, such as brain tissue [16]. Transporting the long-chain fatty acids from the cell cytoplasm to the mitochondrial matrix, where *β*-oxidation of fatty acids and adenosine triphosphate (ATP) production happens, is the main function of the L-carnitine [17]. Acetyl-L-carnitine (ALC) is the acetylated form of L-carnitine synthesized by the enzyme carnitine acetyltransferase. Like L-carnitine, it is available at relatively high levels in the brain [18]. Due to ALC's amphiphilic structure, when exogenously administered, its crosses from the blood-brain barrier are easier than those of free carnitine [19]. Recent evidence indicates that beyond its classical roles in improving the overall energy status of the brain, ALC can have other neuroprotectant effects in the brain, including improving mitochondrial function, protecting the brain from excitotoxicity and neuromodulatory effects, antioxidant and anti-inflammatory activity, and membrane-modifying effects [16]. Due to these multiple mechanisms of action and also considering its favorable safety and tolerability profile, during the last two decades, potential neuroprotective effects of exogenous ALC administration in treating various neurological disorders have been investigated; most notably, its therapeutic benefits on neurological disorders, like Huntington's disease, Alzheimer's disease, major depression, and hepatic encephalopathy, have received much attention [20–23]. Besides, there is growing evidence regarding the possible neuroprotective effects of L-carnitine and its derivatives, like ALC, against cerebral ischemia injury [24, 25].

Considering this evidence, we hypothesized that early treatment with ALC might show clinical benefits in acute ischemic stroke patients. Thus, this pilot clinical trial was undertaken to investigate whether early ALC administration can improve functional and neurological outcomes in patients with acute ischemic stroke. In the present study, we further evaluate the alteration of the serum levels of the inflammatory and oxidative stress biomarkers during treatment with ALC to investigate the clinical effects of ALC in stroke in relation to its anti-inflammatory and antioxidant effects.

## 2. Material and Methods

### 2.1. Study Design and Organization

It was a single-center, double-blind, randomized placebo-controlled pilot clinical trial with a 12-week follow-up, which was carried out in a tertiary referral hospital in the West of Iran from July 2018 until September 2019. The study was registered at the Iranian Registry of Clinical Trials (IRCT; http://www.irct.ir, https://www.irct.ir/trial/32740) on 25 July 2018, with the registration code of “IRCT20150629022965N17.” Information about the study was given to patients or their legally acceptable representatives, and written informed consent forms were obtained from the eligible participants who fulfilled the inclusion and exclusion criteria.

### 2.2. Participants

From July 2018 until September 2019, all cases with a diagnosis of acute ischemic stroke admitted to our hospital were screened by the research team for eligibility for the study. The inclusion criteria for eligibility included the following: (1) men or women with the ages above 18 years; (2) diagnosis of first-ever ischemic stroke; (3) onset of symptoms less than 24 h; (4) measurable neurologic deficit (National Institutes of Health Stroke Scale (NIHSS) score of >3 and <22); and (5) not candidates for thrombolytic therapy and mechanical thrombectomy. The exclusion criteria included the following: (1) severe stroke (NIHSS > 22); (2) hemorrhagic stroke; (3) presence of evidence of other diseases conditions of the central nervous system (CNS), such as demyelinating disorders, brain tumors, previous craniotomy, and severe brain injury; (4) acute or chronic hepatic or renal dysfunction (LFT > 3× upper limit of normal, creatinine > 2 mg/dl); (5) acute or chronic inflammatory or infectious disorders; (6) using any anti-inflammatory and antioxidant supplements other than prescribed medications; (7) history of ALC intolerance or allergy; (8) pregnancy or lactation; (9) psychological, pharmacological, or medical factors interfering with the collection or interpretation of the trial data; (10) likelihood of unavailability for 90-day follow-up; and (11) occurrence of any adverse effects leading to complications or intolerance of patients. The diagnosis of acute ischemic stroke was made through clinical and neurologic examinations and cerebral imaging (head computed tomography (CT) and/or magnetic resonance imaging (MRI)).

### 2.3. Randomization and Blinding Procedure

A total of 69 patients who were satisfied with the trial criteria and had given written informed consent using the block randomization method were allocated to receive either ALC (intervention group; *n* = 34) or a matching placebo (control group; *n* = 35). ALC and placebo were identical in appearance. Randomization was performed by a statistician without any clinical contribution to the trial. Additionally, researchers who administered the treatment and those who assessed the outcomes, as well as all participants, were blinded to the group allocation until the collection of all trial data, analysis of all blood samples, and performing statistical analysis.

### 2.4. Study Intervention

All patients, irrespective of the treatment group, received routine standard of care for acute ischemic stroke based on the accepted medical criteria and treatment guidelines [26]. In addition to standard care, the ALC-treated group received 1000 mg of oral ALC 3 times per day for three consecutive days (a total of nine doses), and participants in the control group received the same dose of a placebo for the same period. A nasogastric tube was used for medication administration in patients with difficulty in swallowing (dysphagia).

### 2.5. Clinical Assessment

Background demographic and clinical characteristics of the participants, including gender, age, BMI, history of smoking, risk factors, and underlying diseases such as diabetes, hyperlipidemia, coronary artery disease, hypertension, and atrial fibrillation, stroke etiology, medication history, and mean time from stroke onset, were recorded from their medical records on admission. Further, relevant laboratory and clinical data of subjects, including triglyceride (TG), total cholesterol (TC), low-density lipoprotein cholesterol (LDL-C), high-density lipoprotein cholesterol (HDL-C), systolic and diastolic blood pressure, and fasting blood glucose (FBS), were also recorded at baseline.

The main efficacy variable was quantifying any neurological shortage at 90 days following stroke using the NIHSS scale, which contains 15 items and is a neurologic examination stroke scale providing a quantitative metric of neurological deficit related to stroke. Each item scores a specific neurological ability between 0 and 4; a score of 0 represents a normal functioning in that specific ability, while a higher score indicates some level of neurological disability. Total scores on the NIHSS ranged from 0 to 42, and higher scores reflect more severe neurological disability [27]. NIHSS was prospectively recorded for every participant on admission and 90 days following the stroke onset. At the time of the patient's admission, the severity of the stroke was classified based on the NIHSS score into three groups: mild stroke with NIHSS score of ≤8, moderate stroke with NIHSS score between 9 and 15, and severe stroke with an NIHSS score of ≥16. The stroke severity on day 90 was also classified as a complete or nearly complete recovery (NIHSS score of 0 to 1), mild (NIHSS score of 2 to 7), moderate (NIHSS score of 8 to 14), and severe (NIHSS score ≥ 15).

The poststroke functional limitations were the second efficacy outcome measure of the trial, which was evaluated by the modified Rankin Scale (mRS) on day 90 [28]. mRS is a validated measure of functional outcome after stroke with potential scores from 0 (indicating no functional limitation) to 5 (indicating severe functional disability); a score of 6 denotes death. An unfavorable functional outcome was defined as mRS score of 2 to 6, and a favorable functional outcome was defined as mRS score of 0 to 1. All efficacy outcomes were determined by a single experienced nurse blinded to the patients' condition and grouping.

To evaluate the safety and tolerability of the study medications, during the active treatment period, investigators closely monitored each patient for evidence of study drug intolerance, and all adverse effects reported spontaneously by the patients or noticed by the investigators were recorded.

### 2.6. Sampling and Biochemical Measurements

Fasting blood samples were taken through an antecubital venipuncture at baseline (before administration of study drug) and at the end of the 3-day treatment protocol. Although the time window for immune-inflammatory assay after stroke remains to be fully understood, the choice of 72-hour for measuring the oxidative stress and inflammation biomarkers was according to the previous literature, reporting that the oxidative stress and inflammatory activity mainly occur during the first 72 hours following an acute ischemic stroke, as high levels of the inflammatory and oxidative stress biomarkers during the acute phase of stroke can predict worse long-term neurological outcomes in these patients [29]. Plasma was separated and kept at −70°C until analysis. The samples were analyzed in a blinded manner by laboratory technicians in duplicate.

In order to evaluate the serum level of TAC, as a total antioxidant capacity, the ferric-reducing ability of plasma (FRAP) method was applied, which was based on the plasma ability for reducing Fe^3+^ to Fe^2+^ by the action of the electron donation from antioxidants, in an acidic medium at the presence of tri-pyridyl triazine (TPTZ) [30]. The DTNB (5,5′-dithio-bis-(2-nitrobenzoic acid)) colorimetric method was used to evaluate the total thiol group (TTG). In this method, there is a reaction between DTNB and the SH groups for producing a yellow-colored product [31]. Glutathione peroxidase (GPx), catalase (CAT), and superoxide dismutase (SOD) activities, as three major enzymatic antioxidant defense systems against oxygen species, were assessed by using spectrophotometric assay kits based on the procedure specified by the manufacturer (Zelbio Co., Germany). Malondialdehyde (MDA), as a secondary product resulting from lipid peroxidation, was measured spectrophotometrically by its reaction with thiobarbituric acid [32]. The serum level of nitric oxide (NO), as a nitrosative stress indicator, was assayed by using the Griess Reagent System according to the measurement of total nitrate in the presence of N-1-ethylenediamine dihydrochloride (NED, Griess reagent) and sulfanilamide. Furthermore, plasma concentrations of IL-6, TNF-*α*, and sICAM-1, as immune-inflammatory markers, neuron-specific enolase (NSE), as a brain neuronal injury indicator, and total L-carnitine levels were determined with commercially available quantitative sandwich ELISA kits, which were obtained from ZellBio GmbH Co., Germany.

### 2.7. Statistical Analysis

All analyses were made by using SPSS software (version 20). The distribution of continuous data was assessed using the Kolmogorov–Smirnov test to determine the use of parametric or nonparametric tests. All analyses were performed in accordance with the intention-to-treat (ITT) principle, defined as all randomized participants taking at least one dose of the trial drug. Missing data were substituted by the other group's mean. The normally distributed data were expressed as mean ± standard deviation (SD) and analyzed using the independent *t*-test. Data with nonnormal distribution were expressed as median (interquartile range (IQR)) and analyzed by the Mann–Whitney *U* test. Categorical variables were expressed as frequency and analyzed by the chi-square or Fisher exact test (if more than 20% of the categories were expected to have a frequency less than 5). Participants' background and the risk factors of interest were entered in the univariate linear regression as explanatory predictors, and two variables of NIHSS and mRS scores 90 days after the intervention were considered dependent variables. Those with a *p* value < 0.20 in the univariate analyses were entered into multivariable analyses. *p* value < 0.05 was considered statistically significant.

## 3. Results

### 3.1. Sample Characteristics

**Figure 1:**
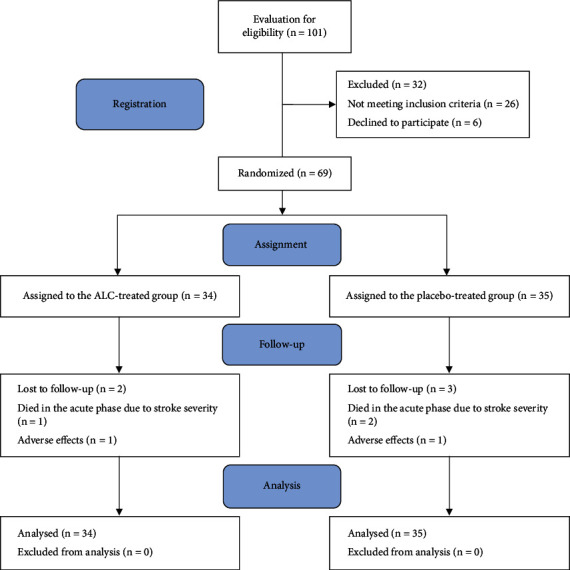
The study flow diagram.

The flow diagram of the study is shown in Figure 1. A total of 101 patients with acute ischemic stroke were screened to determine their eligibility for participation in the trial; of whom, 69 subjects were recruited and randomized into the ALC-treated group (*n* = 34) or the placebo-treated group (*n* = 35). All participants took at least one dose of the trial medication. Ten cases did not complete the trial (four in the ALC group and six in the placebo group). As mentioned above, to prevent the overestimation of efficacy, all analyses were made on an intention-to-treat (ITT) analysis dataset (on 69 participants). Due to less than 20% dropout rates and similar disease courses in the study groups, we replaced missing data with the other group's mean data [33]. The ITT population characteristics at baseline are summarized in Table 1. At baseline, participants in two treatment groups were matched for demographics and laboratory and clinical characteristics. As indicated, the medical history and risk factors did not show relevant group differences. The mean time to treatment was 8 : 31 ± 8 : 57 hours in the subjects treated with ALC and 9 : 42 ± 11 : 41 hours in the subjects treated with placebo, and the two groups were comparable in this regard.

**Figure 2:**
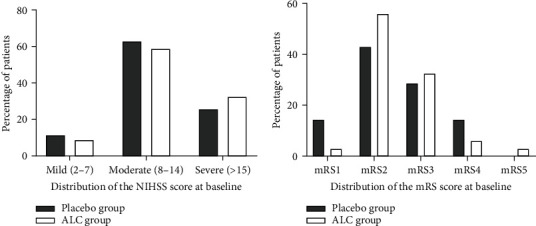
Comparison of the distribution of the NIHSS score (a) and mRS score (b) of the two groups at admission. Note: NIHSS = National Institutes of Health Stroke Scale; mRS = modified Rankin Scale.

The baseline NIHSS scores in the ALC-treated and placebo-treated groups were 13.06 ± 3.70 and 12.26 ± 5.03, respectively. Accordingly, the mean baseline NIHSS score was not significantly different in the two groups (*p* value = 0.45; Table 2). Comparisons of baseline distribution of NIHSS scores by 3-point categories between the study groups also indicated balance in the NIHSS severity at baseline (Figure 2(a)). The study groups were also similar in terms of the mean mRS scores at baseline (2.53 ± 0.78 in the ALC group and 2.51 ± 0.98 in the placebo group; *p* value = 0.62; Table 2). The baseline distribution of mRS scores was also comparable between the two groups (Figure 2(b)). Thus, at baseline, the NIHSS and mRS scores between groups were statistically identical.

### 3.2. Clinical Efficacy Outcomes

**Figure 3:**
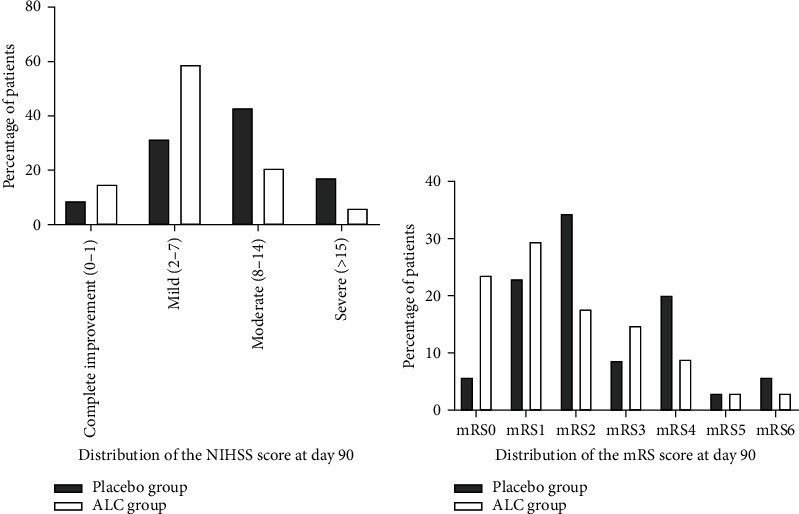
Comparison of the distribution of the NIHSS score (a) and mRS score (b) of the two groups at day 90. Note: NIHSS = National Institutes of Health Stroke Scale; mRS = modified Rankin Scale.

The results indicated that treatment with ALC led to statistically significant reductions in mean NIHSS scores on day 90 compared to the control group, with a follow-up mean NIHSS score of 9.60 ± 4.32 for the placebo group and 7.24 ± 4.39 for the ALC group (Table 2). The mean changes in the NIHSS score on day 90 after stroke in comparison to the baseline were −5.82 ± 4.51 for the ALC group, while it was −2.83 ± 3.08 for the placebo group, which showed a statistically significant difference between the groups (*p* value = 0.002; Table 2). Consistent with the results of the NIHSS, substantial differences were observed between the ALC- and placebo-treated patients on day 90 for the mRS (Table 2). The mean mRS scores on day 90 were significantly lower in the ALC-treated cases than in the placebo-treated cases (1.65 ± 1.6 vs. 2.54 ± 1.60; *p* value = 0.02). The results of the multivariable analyses are presented in Tables 3 and 4 for NIHSS and mRS, respectively. After adjusting for other variables in the model, compared to the patients treated with placebo, those treated with ALC had lower NIHSS score (*β*: -2.40, 95% CI: -0.69, -4.10 (*p* = 0.007)) and mRS score (*β*: -1.18, 95% CI: -0.52, -1.84 (*p* = 0.001)) 90 days after the intervention. The analysis of NIHSS score distribution by 4-point categories at 90 days also showed (Figure 3(a)) that the patients treated with ALC shifted towards a better outcome in comparison to the placebo patients (*p* value = 0.04).  Figure 3(b) represents the distribution analysis in all mRS scores on day 90 after stroke in the two groups, which indicated more improvement in the ALC-treated patients, although this improvement did not represent a significant difference compared to the control group (*p* value = 0.19). On the other hand, the percentage of subjects with a favorable functional outcome (score 0 or 1) on mRS on day 90 was 52.9% in the ALC group and 28.6% in the placebo group, which showed a significant difference (*p* value = 0.05).

### 3.3. Safety and Tolerability

Table 5 represents results related to the most frequent adverse effects. The most frequently reported adverse effects by the participants, irrespective of the treatment group, were gastrointestinal-related symptoms such as nausea, upset stomach, and diarrhea. The reported adverse effects were mild to moderate in severity, and no serious adverse effects were occurred. In each studied group, one patient discontinued the study because of an intolerable adverse effect. As shown in Table 5, when the ALC group was compared with the placebo group, no reported adverse effects were significantly more prevalent in the ALC-treated group. Therefore, the safety outcome indicated an excellent safety profile and ALC tolerance in patients with acute ischemic stroke.

### 3.4. Comparison of Inflammatory and Oxidative Stress Biomarkers between the Two Groups

Table 6 represents the comparison of the changes in the serum levels of inflammatory and oxidative stress biomarkers between the two groups. As shown, the baseline level of inflammatory and oxidative stress markers showed no significant difference between the groups. Based on the results, 72 hours after the drug administration in the cases receiving ALC, serum levels of TAC, SOD, and GPx, as antioxidants biomarkers, were significantly higher than those in the placebo group (*p* value = 0.01, 0.02, and 0.01, respectively). After three days of the treatment protocol, also the serum levels of TNF-*α*, IL-6, and ICAM-1, as proinflammatory cytokines, decreased more in the ALC group than in the placebo group (*p* value = 0.04, 0.02, and 0.03, respectively). Furthermore, on day 3, the mean changes in the serum levels of TNF-*α*, ICAM-1, TAC, NSE, and SOD in the patients receiving ALC were significantly higher than those in the patients receiving placebo. Despite a trend towards more favorable results on serum levels of CAT, TTG, MDA, and NO in the ALC-treated patients in comparison with the placebo-treated patients on day 3, no significant difference was seen between the studied groups in terms of serum values of these factors. Moreover, at the end of the third day of the treatment, the subjects in the ALC group showed a significant increase in the serum level of total L-carnitine compared to the placebo group (*p* value < 0.001).

In summary, ALC supplementation caused a significant decline in the serum levels of TNF-*α*, ICAM-1, IL-6, and NSE and a significant increase in the serum level of SOD, TAC, GPx, and total L-carnitine in the subjects with acute ischemic stroke compared to the placebo treatment.

## 4. Discussion

The present clinical study provided promising evidence that early supplementation with ALC can be useful in patients with acute ischemic stroke who are not eligible for reperfusion therapy and can improve poststroke outcomes concerning neurological deficit and functional disability. The obtained results also revealed that the ALC might exert neuroprotective activity in these patients, at least in part, through its ability to regulate inflammation and oxidative stress responses secondary to brain ischemia.

From a metabolic viewpoint, due to very limited energy storage and high energy requirements, the brain has a high sensitivity to blood flow interruptions [3]. Concerning the energy metabolism and the critical role of mitochondria for cellular energy generation, impairment of mitochondrial energy metabolism during an acute ischemic stroke can lead to overproduction of ROS and oxidative damage to neurocellular biomolecules [34]. Thus, compounds that can promote brain energy and oxygen metabolisms such as L-carnitine and its derivatives can be potential therapeutic strategies in treating brain ischemia. In support of this perspective, there is compelling evidence from experimental and clinical research concerning the crucial impacts of L-carnitine and ALC in improving the energetic state of the neuron in neurological disorders like depression and Alzheimer's disease [20]. Moreover, numerous experimental studies reported promising results regarding the effectiveness of L-carnitine and its derivatives against neuromuscular ischemia-reperfusion damage [35]. The therapeutic potential of ALC on modulation of brain energy status and prevention of neurological dysfunction and/or neuronal injury in animal models of both global and focal cerebral ischemia has also been studied. Rosenthal et al., in their research, showed that ALC administration potentiates normalizing brain energy metabolites and enhances improving neurological outcomes in a clinically related model of global cerebral ischemia and reperfusion [36]. Moreover, another research on the animal model of brain ischemia indicated that ALC administration immediately after ischemia and during reperfusion had a potentially beneficial effect on cerebral ischemia injury. This effect mainly occurs through the mechanisms that lead to quicker improvement and recovery of brain energy production and, as a result, reduction in lactic acid generation in early postischemic reperfusion [37]. Also, promising results from clinical and experimental studies suggested that ALC supplementation protects against traumatic brain injury- (TBI-) induced energy metabolism dysfunction and lactate accumulation [38]. Besides, research on animal and human models showed the beneficial role of ALC in the restoration of ammonia-induced cerebral energy depletion [39, 40].

Despite the role of glucose as the major energy source for the adult brain in normal circumstances, blood levels of ketones and free acetyl-CoA could be crucial for brain energy substrate under some metabolic conditions [41]. It seems that ALC can enhance oxidative cerebral energy production, decrease anaerobic glycolysis, and reduce lactic acidosis by providing acetyl groups for the acetyl-CoA synthesis that can enter the citric acid cycle. Reduction in brain lactic acidosis that significantly affects ROS formation is one of the probable mechanisms through which ALC can exert protective effects on brain tissue against oxidative stress damage [20]. Inducing antioxidant gene expression and shifts in both the mitochondrial and cytosolic redox states may be other probable mechanisms that, through them, ALC can exert antioxidant effects during oxidative stress [42]. Additionally, L-carnitine and ALC, through suppressing oxidative stress in mitochondria and then improving mitochondrial function, have shown antiapoptotic effects [43, 44]. It has been reported that L-carnitine and its derivatives influence the expression of many genes that are implicated in the modulation of the production of free radicals, cellular antioxidant defenses, and restoration and stabilization of mitochondrial actions [45].

It is well established that L-carnitine and its derivatives can act as potent antioxidant agents. Direct scavenging free radicals, maintenance of mitochondria integrity in stress conditions, chelating catalytic metals-promoters of ROS, prevention of ROS formation, inhibition of ROS-generating enzymes, affecting redox signaling through activating nuclear factor erythroid-derived 2-like 2 (Nrf2) and peroxisome proliferator-activated receptor alpha (PPAR*α*) and inhibition of nuclear factor-kappa enhancer of B cells (NF-*κ*B), regulation of vitagenes, and synthesis of heat shock proteins (HSPs), thioredoxins, sirtuins, and other antioxidant molecules are several pathways that L-carnitine and its derivatives through them can exhibit their antioxidant impacts [46]. Several studies have investigated the modulatory effects of L-carnitine and ALC on oxidative stress parameters in neurological conditions. In this regard, Zydan et al., in an animal model of the relapsing-remitting experimental autoimmune encephalomyelitis, showed that ALC as a supplementary treatment to dexamethasone results in a significant reduction in MDA and caspase-3 activity and a significant elevation in Bcl-2 and GSH expression and consequently provides considerable antiapoptotic and antioxidant milieu in the spinal cord and brain milieu [47]. In another study, L-carnitine in vitro via scavenging oxygen free radicals, preventing lipid peroxidation, augmenting antioxidant defense, inhibiting cell apoptosis, and regulating apoptosis-related gene expression exerts protective effects against oxidative injury of neuronal cells [48]. Liu et al. also, in their study in a postcardiac arrest dog model, observed that ALC treatment could significantly reduce the brain level of protein carbonyl groups that are oxidative tissue damage biomarkers. Moreover, the results of this study also revealed that the antioxidant influence of ALC on brain tissue might be related to its ameliorative effect on tissue lactic acidosis [49]. In another report, the addition of ALC to astrocytes by induction of the antioxidant enzyme heme oxygenase-1 showed protective effects against the brain oxidative injury induced by inflammatory cytokine insult [50]. Several studies have also compared the antioxidant properties of the ALC and L-carnitine. In this respect, the results of one experimental study in rats revealed that although L-carnitine and ALC showed comparable effects in the increment of carnitine levels in the brain and blood, ALC had higher efficacy in the reduction of oxidative brain injury [51]. Consistent with these results, another in vitro research showed that although the pretreatment with L-carnitine and ALC can reduce oxygen-glucose deprivation-induced cell damage by augmenting the activities of ATPase and SOD, only ALC can exert a protective impact on neuronal cell damage following ischemia in vivo. The different affinity of L-carnitine and ALC to the organic cation/carnitine transporter system on astrocytes that is important for uptake of ALC and L-carnitine in these cells might influence the neuroprotective effects of these agents against ischemic damage [52]. The impact of ALC and L-carnitine on oxidative stress mediators was also studied in some clinical research. It has been reported that the treatment with ALC in patients with active multiple sclerosis was associated with reduced cerebrospinal fluid levels of NO, reactive metabolites, protein nitration, elevated GSH content, and increased GSH/GSSG ratio [53]. The results of another study on patients with pemphigus vulgaris also revealed that L-carnitine administration significantly increased the serum levels of TAC and carnitine and significantly decreased oxidative stress index compared to the placebo treatment in these patients [54]. Beneficial impacts of L-carnitine treatment on antioxidant enzyme activities and oxidative stress mediators in cases with coronary artery disease have also been reported [55]. Consistent with this clinical evidence, our findings also provided further clinical evidence in terms of modulatory effects of ALC on oxidative stress milieu in acute ischemic stroke.

With the concept of the important role of neuroinflammation in neuronal injury after cerebral ischemia, it seems that inflammation may be another target where anti-inflammatory compounds such as ALC can exert their benefits against ischemic injury. There is an interrelation between inflammation and oxidative stress, one of which can easily be augmented by another. Oxidative stress, by inducing activation of transcription factors, like NF-*κ*B, enhances the generation of various proinflammatory mediators that intensify neuronal damage [56]. It was found that the antioxidants agents, like L-carnitine and its derivatives by suppression of the NF-*κ*B pathway, can exert anti-inflammatory effects in pathological conditions such as ischemic stroke [57]. The regulatory impacts of ALC and L-carnitine on inflammatory mediators in various pathological inflammatory conditions have been shown in previous research. In this regard, it has been shown that administration of L-carnitine at a dose of 1000 mg/d for 12 weeks in cases with coronary artery disease causes a significant reduction in the level of inflammatory biomarkers such as TNF-*α* and IL-6. This research also showed a relationship between the level of inflammatory biomarkers and antioxidant status after L-carnitine supplementation conveying that the anti-inflammatory properties of L-carnitine might be related to its antioxidant properties [58]. The combined application of ALC+quercetin in an animal model of Alzheimer's disease also significantly reduced hippocampus levels of inflammatory biomarkers, like TNF-*α* and IL-6 [59]. Consistent with these results, in the present study, it was found that ALC as a major metabolite of L-carnitine significantly reduced the serum levels of the proinflammatory cytokine, like TNF-*α* and IL-6.

Extensive research has shown that overexpression of adhesion molecules, like VCAM-1 and ICAM-1, on the cerebral endothelium by recruiting circulating leukocytes into the inflammation areas has a crucial role in inflammation and progress of ischemic damage following acute stroke [60]. Preliminary evidence indicated that L-carnitine and its metabolites by inhibitory effects on expressing adhesion molecules, like ICAM-1 during the inflammatory process, such as coronary heart diseases, can exhibit a myocardial protection effect [61]. In agreement with this view, in the present study, we also observed that ALC therapy led to a significant decline in serum levels of proinflammatory mediators, like ICAM-1.

Releasing excitatory amino acids, like glutamate, to extracellular space is excessively triggered by interruption or severe attenuation of blood flow in the infarcted region of the brain after stroke. Glutamate receptor overactivation leads to several detrimental results, such as cellular calcium homeostasis impairment, free radical overproduction, and oxidative toxic stress induction, increasing mitochondrial permeability transition, triggering several transcription factors and their gene expression and secondary excitotoxicity. All these mechanisms synergistically induce or aggravate neuronal damage [62]. Preliminary evidence indicated that L-carnitine and its acetylated form (ALC) by direct antagonism of glutamate receptors, stimulation of metabotropic glutamate receptors, inhibition presynaptic of glutamate release, and stimulation of gamma-aminobutyric acid (GABA) receptors can exhibit a protective effect against glutamate neurotoxicity [63, 64]. In this way, in one experimental research, the presence of ALC together with N-methyl-D-aspartate (NMDA) in cultured cortical neurons showed protective effects against NMDA-induced acute and delayed neuronal death [42]. In a similar vein, in another study in vitro, chronic exposure to ALC inhibited hippocampal neuronal cell death in reaction to glutamate exposure [64]. Also, the findings of one experimental research on the newborn rat model of hypoxia-ischemia showed that the treatment with carnitine could prevent the increase in the excitatory amino acid levels, such as glutamate, that occurs during cerebral ischemia [24]. Therefore, this evidence indicates that the neuroprotective impacts of L-carnitine and its derivatives on cerebral ischemic damage may also be mediated by their regulatory effects on the hemostasis of excitatory neurotransmitters such as glutamate.

In summary, at least in theory, L-carnitine and its derivatives could act as a neuroprotective agent through several mechanisms in ischemic stroke. Supporting this hypothesis, some published clinical research has addressed the efficacy of L-carnitine and its derivatives in treating ischemic stroke. In this regard, Chichanovskaya et al., in their recent study, found that L-carnitine administration in patients with ischemic stroke in the early period of rehabilitation resulted in the reduction of the intensity of neurological impairment on the NIHSS scale and also reduced the percentage of patients who needed help according to the Barthel Index [65]. In Postiglione et al.'s study, in patients suffering from chronic cerebrovascular diseases, it was found that a single high-dose (1.5 g) injection of ALC can enhance cerebral blood flow in these patients compared to the placebo [66]. Moreover, Kazemian et al.'s study demonstrated that L-carnitine, in combination with fat emulsion, is neuroprotective in patients with acute ischemic stroke [67].

A known favorable safety profile is another property of ALC that makes it a potentially useful neuroprotective treatment for treating patients with stroke. Previous studies have shown that the ALC was well tolerated by the patients and showed comparable tolerability to the placebo treatment [23]. Therefore, in light of current evidence on the efficacy as well as the safety profile of ALC, its use in treating acute ischemic stroke as an adjunct to current standard management seems rational.

Despite the novelty of the findings of the present study, the following limitations should be taken into consideration. First, the sample size was relatively small, and this was a single-center investigation. Another limitation was using a relatively moderate dose of ALC and the relatively short therapy period. Third, to assess the influence of ALC on the serum levels of biochemical biomarkers in the acute phase of the stroke, only two samples were taken from each patient at baseline and 72 hours followed by the treatment because of structural constraints. Additionally, we only selected the specific proinflammatory mediators that have been mostly addressed in the literature regarding their impacts on stroke's clinical outcomes. Therefore, further studies are required for a deeper insight into the mechanistic pathways of ALC in acute ischemic stroke. Additionally, in the present study, the ALC was administered within 24 hours after the onset of stroke symptoms; elucidation of the therapeutic window of ALC, which could confer neuroprotection in stroke patients, should be taken into account in future studies.

## 5. Conclusion

Our pilot clinical trial provided promising evidence that early ALC administration may be encouraging in stroke patients who are not candidates for reperfusion therapy. Part of the neuroprotective effects of ALC may be explained by its regulatory effect on the inflammatory and oxidative stress reactions occurring in the brain after ischemia. However, further studies are required before supplementation with ALC can be considered for the treatment of patients with acute ischemic stroke.

## Figures and Tables

**Table 1:** Baseline demographics and clinical features of the intention-to-treat population.

Variable	ALC group (34 patients)	Placebo group (35 patients)	*p* value
Age (years), mean ± SD	65.24 ± 12.89	70.37 ± 13.58	0.11
BMI (kg/m^2^)	24.25 ± 2.96	25.29 ± 3.31	0.28
Sex (M/F), *n* (%)	19/15 (55.9/44.1)	13/22 (37.1/62.9)	0.15
Prevalence of risk factors			
Smokers, *n* (%)	17 (50.0)	13 (31.7)	0.33
Hypertension, *n* (%)	24 (70.6)	23 (65.7)	0.79
Diabetes, *n* (%)	10 (29.4)	11 (31.4)	1.00
CAD, *n* (%)	11 (32.4)	6 (17.1)	0.17
Hypercholesterolemia, *n* (%)	11 (32.4)	6 (17.1)	0.17
Other factors, *n* (%)	10 (29.4)	7 (20.0)	0.41
SBP (mmHg), mean ± SD	153.24 ± 34.96	153.43 ± 26.34	0.97
DHP (mmHg), mean ± SD	87.35 ± 16.75	84.00 ± 11.16	0.33
Total cholesterol (mg/dl), mean ± SD	196.26 ± 21.21	192.40 ± 18.36	0.42
LDL cholesterol (mg/dl), mean ± SD	135.32 ± 25.49	129.57 ± 18.08	0.28
HDL cholesterol (mg/dl), mean ± SD	39.97 ± 4.6	41.02 ± 4.99	0.36
TG (mg/dl), mean ± SD	156.41 ± 21.34	148.74 ± 21.76	0.14
FBS (mg/dl), mean ± SD	106.82 ± 28.03	112.62 ± 25.06	0.36
Medications prior to stroke			
Antiplatelet treatment, *n* (%)	18 (52.9)	12 (34.3)	0.14
ACEI treatment, *n* (%)	16 (47.1)	19 (54.3)	0.63
ARB treatment, *n* (%)	9 (26.5)	6 (17.1)	0.31
Beta-blocker treatment, *n* (%)	12 (35.3)	10 (28.6)	0.61
CCB treatment, *n* (%)	9 (26.5)	7 (20.0)	0.57
Diuretic treatment, *n* (%)	6 (17.6)	7 (20.0)	1.00
Insulin with oral antidiabetic treatment, *n* (%)	7 (20.6)	4 (11.4)	0.34
Oral antidiabetic treatment, *n* (%)	9 (26.5)	7 (20.0)	0.57
Statin treatment, *n* (%)	18 (52.9)	20 (57.1)	0.81
Anticoagulant treatment, *n* (%)	6 (17.6)	5 (14.3)	0.75
Cause of stroke			
Atheromatosis, *n* (%)	16 (47.1)	15 (42.9)	0.95
Embolus, *n* (%)	7 (20.6)	7 (20.0)	
Lacunar infract, *n* (%)	5 (17.4)	6 (17.1)	
Other causes, *n* (%)	3 (8.8)	2 (5.7)	
Undetermined, *n* (%)	3 (8.8)	5 (14.3)	
Time to treatment after onset of stroke (h), mean ± SD	8 : 31 ± 8 : 57	9 : 42 ± 11 : 41	0.63

Note: ALC = acetyl-L-carnitine; BMI = body mass index; SD = standard deviation; DHP = diastolic blood pressure; CAD = coronary artery disease; SBP = systolic blood pressure; HDL = high-density lipoprotein; TG = triglycerides; LDL = low-density lipoprotein; ACEI = angiotensin-converting enzyme inhibitors; FBS = fasting blood sugar; ARB = angiotensin II receptor blockers.

**Table 2:** Comparison of the changes of the NIHSS and mRS scores between 2 groups at admission and 3 months after stroke.

Variable	ALC group (*n* = 34)	Placebo group (*n* = 35)	*p* value
Admission NIHSS score, mean ± SD	13.06 ± 3.70	12.26 ± 5.03	0.45

3-month after-stroke NIHSS score, mean ± SD	7.24 ± 4.39	9.60 ± 4.32	**0.02**

NIHSS alteration from admission to 3 months after stroke, mean ± SD	−5.82 ± 4.51	−2.83 ± 3.08	**0.002**

Admission mRS score, mean ± SD	2.53 ± 0.78	2.51 ± 0.98	0.62

3-month after-stroke mRS score, mean ± SD	1.65 ± 1.6	2.54 ± 1.60	**0.02**

mRS alteration from baseline to 3 months after stroke, mean ± SD	−0.94 ± 1.30	0.11 ± 1.34	**0.002**

Note: ALC = acetyl-L-carnitine; NIHSS = National Institutes of Health Stroke Scale; mRS = modified Rankin Scale. The significant *p* value is shown in bold type.

**Table 3:** The multivariable linear regression analysis for assessing predictors of NIHSS.

Variable		*β*	95% CI	*p* value^∗^
Group	Placebo group	Reference		
	ALC group	-2.40	-0.69, -4.10	**0.007**
Age, years		0.06	-0.003, 0.13	0.06
Diabetes	No	Reference		
	Yes	1.68	-0.14, 3.49	0.07
NIHSS before		0.49	0.29, 0.69	**<0.001**

Note: ALC = acetyl-L-carnitine. ^∗^Adjusted for other variables in the model. The significant *p* value is shown in bold type.

**Table 4:** The multivariable linear regression analysis for assessing predictors of mRS.

Variable		*β*	95% CI	*p* value^∗^
Group	Placebo group	Reference		
	ALC group	-1.18	-0.52, -1.84	**0.001**
Gender	Female	Reference		
	Male	0.38	-0.17, 1.14	0.20
Coronary artery diseases	No	Reference		
	Yes	0.65	-0.12, 1.42	0.098
mRS before		0.87	0.49, 1.27	**<0.001**

Note: ALC = acetyl-L-carnitine. ^∗^Adjusted for other variables in the model. The significant *p* value is shown in bold type.

**Table 5:** Comparison of the frequency of adverse effects related to medication between the 2 groups.

Adverse effects, *N* (%)	Treatment group		*p* value
	ALC group (*N* = 34)	Placebo group (*N* = 35)	
Nausea	7 (20.6%)	3 (8.6%)	0.19
Dyspepsia	5 (14.7%)	2(5.7%)	0.26
Headache	4 (11.8%)	2 (5.7%)	0.43
Diarrhea	2 (5.9%)	0 (0.0%)	0.24

Note: ALC = acetyl-L-carnitine.

**Table 6:** Comparison of the changes of the serum levels of inflammatory and oxidative stress biomarkers at baseline and 3 days after treatment between the 2 groups.

Variable	Group	Baseline, mean ± SD	72 h after treatment	Mean difference	*p* value
CAT	ALC	642.19 ± 95.54	675.61 ± 82.51	33.41 ± 94.48	**0.04**
	Placebo	632.51 ± 103.66	641.37 ± 111.38	8.85 ± 122.64	0.67
	*p* value	0.68	0.15	0.35	—
GPX	ALC	11.94 ± 7.02	15.43 ± 4.85	3.48 ± 8.52	**0.02**
	Placebo	11.57 ± 6.40	12.40 ± 5.39	0.83 ± 3.61	0.18
	*p* value	0.81	**0.01**	0.09	—
MDA	ALC	55.25 ± 18.05	54.20 ± 15.22	−1.04 ± 18.10	0.74
	Placebo	56.40 ± 20.04	56.12 ± 15.76	−0.27 ± 14.78	0.91
	*p* value	0.80	0.60	0.84	—
NO	ALC	70.18 ± 12.47	66.48 ± 11.61	−3.70 ± 10.33	**0.04**
	Placebo	72.10 ± 11.66	69.94 ± 10.33	−2.16 ± 9.30	0.17
	*p* value	0.51	0.19	0.51	—
SOD	ALC	1.00 ± 0.48	1.40 ± 0.62	0.39 ± 0.61	**<0.001**
	Placebo	0.97 ± 0.50	1.04 ± 0.51	0.06 ± 0.46	0.40
	*p* value	0.80	**0.01**	**0.01**	—
TAC	ALC	107.35 ± 46.52	152.90 ± 62.34	45.54 ± 71.76	**<0.001**
	Placebo	111.88 ± 51.29	122.56 ± 49.05	10.68 ± 70.96	0.37
	*p* value	0.70	**0.02**	**0.04**	—
TTG	ALC	296.61 ± 170.61	352.76 ± 175.43	56.15 ± 197.96	0.10
	Placebo	313.64 ± 149.07	335.23 ± 195.39	21.58 ± 172.43	0.46
	*p* value	0.66	0.69	0.44	—
IL-6	ALC	20.89 ± 6.68	16.67 ± 8.34	−4.21 ± 10.52	**0.02**
	Placebo	23.05 ± 4.68	20.89 ± 6.98	−2.16 ± 7.31	0.08
	*p* value	0.12	**0.02**	0.35	—
NSE	ALC	15.53 ± 4.28	13.44 ± 2.80	−2.08 ± 3.82	**<0.001**
	Placebo	16.18 ± 12.63	16.43 ± 15.42	0.24 ± 5.07	0.77
	*p* value	0.77	0.27	**0.03**	—
TNF-*α*	ALC	16.23 ± 7.39	10.48 ± 5.85	−5.57 ± 9.06	**0.001**
	Placebo	16.50 ± 15.19	15.85 ± 13.88	−0.64 ± 4.78	0.43
	*p* value	0.92	**0.04**	**<0.001**	—
ICAM-1	ALC	3.50 ± 1.05	2.58 ± 0.89	−0.93 ± 1.69	**0.001**
	Placebo	3.20 ± 1.24	3.15 ± 1.06	−0.05 ± 0.86	0.72
	*p* value	0.28	**0.03**	**0.003**	—
Total L-carnitine	ALC	5.97 ± 0.99	7.65 ± 2.53	1.67 ± 2.42	**<0.001**
	Placebo	6.50 ± 1.65	6.55 ± 1.70	0.05 ± 1.32	0.81
	*p* value	0.11	**0.03**	**<0.001**	—

Note: ALC = acetyl-L-carnitine; SD = standard deviation; TTG = total thiol groups; TAC = total antioxidant capacity; MDA = malondialdehyde; NO = nitric oxide; CAT = catalase activity; GPX = glutathione peroxidase; TNF = tumor necrosis factor-alpha; SOD = superoxide dismutase; IL6 = interleukin-6; ICAM-1 = intercellular adhesion molecule-1; NSE = neuron-specific enolase. The significant *p* value is shown in bold type.

## Data Availability

The datasets used and analyzed during the current study are available from the corresponding author on reasonable request up to 2 years after publication.

## Abbreviations

ROS: Reactive oxygen species
TNF-*α*: Tumor necrosis factor-*α*
IL-1: Interleukin-1
IL-6: Interleukin-6
MMPs: Matrix metalloproteinases
CAMs: Cell adhesion molecules
ATP: Adenosine triphosphate
ALC: Acetyl-L-carnitine
NIHSS: National Institutes of Health Stroke Scale
CNS: Central nervous system
CT: Computed tomography
MRI: Magnetic resonance imaging
TG: Triglyceride
TC: Total cholesterol
LDL-C: Low-density lipoprotein cholesterol
HDL-C: High-density lipoprotein cholesterol
FBS: Fasting blood glucose
mRS: Modified Rankin Scale
FRAP: Ferric-reducing ability of plasma
TPTZ: Tri-pyridyl triazine
DTNB: 5,5′-Dithio-bis-(2-nitrobenzoic acid)
TTG: Total thiol group
GPx: Glutathione peroxidase
CAT: Catalase
SOD: Superoxide dismutase
MDA: Malondialdehyde
NO: Nitric oxide
NED: N-1-Ethylenediamine dihydrochloride
NSE: Neuron-specific enolase
SD: Standard deviation
IQR: Interquartile range
ITT: Intention-to-treat
Nrf2: Nuclear factor erythroid-derived 2-like 2
PPAR*α*: Peroxisome proliferator-activated receptor alpha
NF-*κ*B: Nuclear factor-kappa enhancer of B cells
HSPs: Heat shock proteins
GABA: Gamma-aminobutyric acid
NMDA: N-Methyl-D-aspartate.
